# Chicago Society of Physicians and Surgeons

**Published:** 1873-04-01

**Authors:** 


### Society Reports.

### CHICAGO SOCIETY OF PHYSICIANS AND SURGEONS.

PROCEEDINGS OF MEETING HELD MARCH 10TH, 1873.

Society called to order as usual, the President, Dr. Trimble, occupying the chair.

The minutes of the previous meeting were read and approved.

Dr. Montgomery submitted a paper upon "Pathology of Ovarian Cystic Tumors;" also, giving several extracts in detail, from the late journals of the country, and the advancement made upon this subject during the last twenty-five years. He spoke at length upon the particular kind of cell (that is, when found), and called by Dr. Atlee "the ovarian granular cell" in the cysts of ovarian tumors, called by Laenec "the corpuscles inflammatories." This cell is described as being of a grayish-brown color, and varies from one-eight thousandths to one-seven hundreths of an inch in diameter, and was found in thirty-one out of thirty-four specimens examined by Dr. Atlee. This cell is pathognomonic of ovarian tumors and no other. In dropsy of the broad ligaments, a tumor, that forms the most close resemblance possible to ovarian tumors, this peculiar cell has not been found; nor in fibro-cystic tumors of the uterus.

By request of the Society, Dr. Trimble read a paper on "Tobacco." After giving a brief history of its discovery by Europeans, and its introduction into use in Europe, he gave a brief account of its natural, commercial, and chemical history, and its application to disease; and remarked, though other remedies had very nearly banished it as a therapeutic agent, yet it has strong and active medicinal powers. He stated that, by analysis, it had been found to contain, among other constituents, nicotine, hydrocyanic acid, and an empyreumatic oil, all of which were most virulent poisons; and asked : "How, then, can a plant containing three such deadly and active poisons, be used so freely, without great detriment to the health? In addition to its sedative and narcotic powers, tobacco is emetic, cathartic, and sialagogue." He thought that it operates through the nervous system, and through the circulation. Though its effect, when moderately used, was to allay nervous irritability, and to produce a state of languor and repose, yet in larger quantities it often occasioned confusion of the mind, vertigo, stupor, faintness, nausea, vomiting, and general depression of the nervous system and circulation ; its toxicological effect being continued and distressing nausea, feeble pulse, cool skin, syncope, convulsions, and death. He spoke especially of its injurious effects upon the young and sedentary, and of the liability of such to resort to alcoholic stimulants to relieve the depression and prostration caused by it. He then proceeded to state some of the evil effects produced by its use, and quoted numerous authors to substantiate his remarks. Nervous excitement, insomnia, emaciation, general debility, indigestion—producing in some a diminution of the amount of fæces, and constipation—were some of its lesser evils; while its effect upon the heart's action was, in some individuals, very marked, producing what had been termed narcotism of the heart, irritation of the gums and lips, in some instances terminating in cancer, several cases of which he had seen, and always in men, and those who smoked tobacco. It has been known to affect the external senses, and deafness and amaurosis have been some of its results. He quoted cases to show that it produced baldness. He also quoted from a work by Dr. Napheys, to show that it destroyed, at a comparatively early age, the virility of men, and was supposed to be one of the great causes of the decrease of births in our American population. In conclusion, he remarks: "These are some of the evil effects of this most potent drug, whose use has become an almost universal infatuation in this western hemisphere, and whose nauseous fumes and poisonous vapors, pollute the atmosphere of our houses, our public halls, our clothing, our breaths, and the very air we breathe. As a medicinal agent we can readily substitute other substances for it; and for all other purposes, it would be a blessed boon to mankind if it was banished from the face of the earth. The good Queen Victoria, it is said, prohibits the use in her palaces and privote domains. If I had the power, I would make of it one great holocaust—one grand and final *smoke.* "

Dr. Hyde thought the use of tobacco preserved the teeth, as he had been so assured by dentists who had investigated the subject; and, as a general thing, teeth were better preserved in the male than in the female sex ; also, that there were less nervous diseases associated with men than women. He quoted from Dr. Trousseau, who says tobacco is undoubtedly a cathartic, and does not produce constipation. He also quoted from Dr. Hammond, who says his weight (Dr. Hammond's) is increased when he smokes. Dr. Hyde thought the truth lies between the two extremes, both with regard to the use of tobacco and alcohol, by taking too much of either. He thought the loss of hair was more attributable to a dry climate than to the use of tobacco, as in Sweden the people had better heads of hair than those who lived in California, Spain, France and Portugal ; and that it was due to the climate not being so dry in Sweden as in the latter countries ; and the people smoked just as much in one as in any of the other countries.

Dr. Emmons said the bowels became constipated when patients became debilitated and nervous from the use of tobacco. He thinks smoking is more injurious than chewing, and says any one addicted to smoking is quite apt to drink alcoholic liquors, from the reason that if any one smokes too much, and become nervous, they take a drink of liquor to invigorate themselves and steady their nerves; but thinks the moisture of the atmosphere, when one smokes, makes one more nervous than the nicotine in the tobacco does. He said, if a dog was poisoned with nicotine, and recovered, it lost its hair. This, in the lower animals, would go to prove Dr. Trimble's statement. They also have ulceration of the eye-lids, and inflammation of the bulbs is quite prone to spring up.

Dr. Lyman thinks the hair grows better in the tropics; and the largest and finest, and best developed hair is more often found here than in any other zone of the earth.

Dr. Fisher thinks, any one using tobacco uses too much. So with alcohol. Said chewing gave some patients dyspepsia, although the bowels were excited, due to the peristaltic motion being started. He thinks one never ought to learn the habit of smoking or chewing before they attain maturity; but does not advise any one, at this age, to do so, as in young people it produces palpitation, and its use is detrimental. He thinks, after manhood, to smoke moderately is not detrimental, and to abandon it would injure one, *i. e.*, where they have used it moderately. He thinks persons of bilious temperament are less liable to become injured by smoking than others.

Dr. Montgomery thought the use of tobacco caused, in a good many persons, cardiac palpitation, and acted more as a cathartic than the opposite. It also produced dyspepsia, or indigestion, with acid stomach; and to use it in excess produced nervous prostration, and, in severe cases, paralysis. Related a case in which a gentleman had often tried smoking cigarettes, but it invariably nauseated him, although the other male members of the family used them, and the forefathers on each side of the family. It was liable to produce *gastric ulcer*, and was a powerful nerve sedative; and if used to excess it was classed narcotic; and who can be persuaded to think otherwise, when tobacco, analyzed, contains nicotine, or *nicotia*, hydrocyanic acid, sulphureted hydrogen, etc., *some* of the most powerful narcotics we have. Tobacco is hardly ever prescribed, while, on the other hand, alcohol is. So, too, with strychnia, arsenious acid, both valuable medicines. Even theine and caffeine are injurious, if taken in excessive amounts.

